# The epidemiology of *Rickettsia felis* infecting fleas of companion animals in eastern Australia

**DOI:** 10.1186/s13071-018-2737-4

**Published:** 2018-03-20

**Authors:** Yen Thon Teoh, Sze Fui Hii, Stephen Graves, Robert Rees, John Stenos, Rebecca Justine Traub

**Affiliations:** 1 Faculty of Veterinary and Agricultural Sciences, the University of Melbourne, Parkville, VIC 3052 Australia; 2 The Australian Rickettsial Reference Laboratory, Geelong, VIC 3220 Australia

**Keywords:** *Rickettsia*, *Rickettsia felis*, *Ctenocephalides felis*, Australia, Temperature

## Abstract

**Background:**

Flea-borne spotted fever (FBSF) caused by *Rickettsia felis* is an arthropod-borne zoonosis. This study aimed to determine the prevalence, primary species and genotype(s) of *R. felis* infecting fleas from dogs and cats.

**Results:**

All fleas were identified as *Ctenocephalides felis felis*. All rickettsial DNA detected in fleas was identified as being 100% homologous to *R. felis* URRWXCal2, with positivity within tropical, subtropical and temperate regions noted at 6.7%, 13.2% and 15.5%, respectively. Toy/small breed dogs were found to be at a lower odds of harboring *R. felis*-positive fleas compared with large breed dogs on univariate analysis, while DMH and pedigree breed cats were at a lower odds compared to DSH cats. Cooler minimum temperature ranges of between 15 to 20 °C and between 8 to 15 °C increased the odds of *R. felis* positivity in fleas, as did a constrained maximum temperature range of between 27 to 30 °C on multivariable analysis.

**Conclusions:**

Environmental temperature may play a part in influencing *R. felis* prevalence and infectivity within its flea host. Regional climatic differences need to be considered when approaching public health risk mitigation strategies for FBSF.

## Background

*Rickettsia felis* is a bacterial pathogen responsible for FBSF, also known as cat flea typhus (CFT), in humans. Infection results from transmission through fecal contamination of the bite site from an infected flea with the resulting condition typically characterized by a series of non-specific symptoms including pyrexia, maculopapular rash, eschar, myalgia, arthralgia, headache and fatigue [1].

A number of regionally distinct *R. felis*-like species and genotypes have recently been characterized globally and shown to favor specific endosymbiotic relationships with different arthropod species. For example, *Rickettsia* sp. genotype RF2125 preferentially infects *Ctenocephalides felis orientis* and *Ctenocephalides felis strongylus* fleas parasitizing dogs in India [2] and Georgia, USA [3], respectively, whereas *Rickettsia felis* strain LSU is found in non-pathogenic booklice in the United Kingdom and Czech Republic [4]. These *R. felis*-like species and genotypes appear to form a single clade within the genus *Rickettsia* [5]. To date, the only genotype proven to cause zoonotic FBSF is URRWXCal2 [6], for which *Ctenocephalides felis felis* is its flea vector [7]. In parts of Africa, however, *R. felis* URRWXCal2 within *Anopheles* mosquitoes and other *R. felis*-like genotypes have been implicated in cases of fevers of unknown origin [5].

In Australia, FBSF is considered an emerging zoonosis of increasing importance. Recently, cases of FBSF affecting clinically ill patients in Australia were misdiagnosed [8] and *R. felis* exposure was demonstrated in 16% of healthy Australian veterinarians with age and geographic location noted as primary risk factors for exposure. *Rickettsia felis* was detected in 36% of fleas isolated from dogs from regional centers in Western Australia [9] and *R. felis* URRWXCal2 was detected in 19% of flea pools collected from cats in Sydney, Melbourne and Brisbane [10]. In addition, *R. felis* was detected by PCR in the blood of 9% of shelter dogs in Southeast Queensland and 2.3% of indigenous community dogs in the Northern Territory [11, 12], implicating them as potential natural mammalian reservoirs.

Given the growing significance of *R. felis* in Australia, the aim of this study was to ascertain the prevalence, primary species and genotype(s) of *R. felis* infecting fleas isolated from dogs and cats in coastal eastern Australia. In our previously published study, veterinarians from temperate, cooler regions of south-eastern Australia were found to be at significantly higher odds of exposure to *R. felis* than their counterparts in warmer regions [13]. We therefore hypothesize that geographical or climatic variables influence *R. felis* infection rates in fleas, which in turn could influence transmission risk to humans across coastal eastern Australia.

## Methods

### Sample collection

Collection spanned the months from December 2013 to July 2014, a period including summer, autumn and the beginning of the winter months in the Southern Hemisphere. Fleas and host animal data including location, breed, age, sex and infestation load were obtained at periodic intervals from client-owned animals as part of a multi-center field study conducted in dogs and cats across the east coast of Australia by Bayer Animal Health, Australia. Locations were grouped according to climate, with Cairns representing a tropical climate; Ipswich, the Gold Coast and Ballina representing a subtropical climate; and the Central Coast NSW, the Northern Beaches, and Sydney representing a temperate climate.

Animals were broadly grouped by assumed breed characteristics: Chihuahua, Cocker Spaniel, Dachshund, Fox Terrier (including miniature), Jack Russel Terrier, Maltese Terrier, Pomeranian, Pug, Shih Tzu, and Toy Poodle dogs were grouped as “Toy/small breed dog”; Bull Terrier, Bull Arab, Border Collie, Australian Cattle Dog, Kelpie, German Shepherd, Dogue de Bordeaux, Great Dane, Greyhound, Mastiff, Rhodesian Ridgeback, Rottweiler, Tibetan Spaniel, Labrador Retriever and Sharpei dogs were grouped as “Large breed dog”; Bengal, Birman, Burmese, Maine Coon, Himalayan, Persian, Ragdoll, Siamese and Tonkinese cats were represented within the “Pedigree breed cat” grouping.

### Flea identification and DNA extraction

Fleas were identified using diagnostic morphological features [2]. To remove traces of ethanol, fleas were rinsed and vortexed with 300 μl PBS. After being soaked with a further 300 μl PBS for 4 h, fleas were removed from the liquid and a plastic pestle was used to crush each flea individually.

DNA extraction was performed using a Bioline ISOLATE II Genomic DNA extraction kit according to recommended manufacturer’s protocol, and the quality was superficially assessed using a NanoDrop ND1000 (ThermoFisher Scientific, Waltham, MA, USA) spectrophotometer.

### Polymerase chain reaction

Positive cultures of *R. felis* obtained from the Australian Rickettsial Reference Laboratory (ARRL) were used as a positive control, and sterile water was used as a negative control. A previously described qPCR protocol targeting a part of the *glt*A gene was used to screen samples for rickettsiae [14].

Positive samples were subjected to conventional PCR targeting the *glt*A and *omp*B genes using previously described protocols (Table 1) [12]. All positive samples were subject to bidirectional DNA sequencing (Macrogen, Seoul, Republic of Korea).

**Table 1:** Primers used for conventional PCR amplification of partial regions of *glt*A and *omp*B genes [12]

Primer	Sequence (5'-3')	Product size (bp)
*omp*B-F	CGACGTTAACGGTTTCTCATTCT	252
*omp*B-R	ACCGGTTTCTTTGTAGTTTTCGTC	
*glt*A-F1	GCAAGTATTGGTGAGGATGTAATC	654
*glt*A-R1	CTGCGGCACGTGGGTCATAG	
*glt*A-F2	GCGACATCGAGGATATGACAT	
*glt*A-R2	GGAATATTCTCAGAACTACCG	

### Weather data

Weather data (minimum daily temperature, maximum daily temperature, daily rainfall) was obtained from the Bureau of Meteorology Weather Data Services [15]. Data from the closest weather station with records spanning the week before to the date of flea sampling were utilized in the study.

### Data analysis

Data was analyzed using the R statistical software environment [16]. The average temperature of the week preceding the flea-collection was used for analysis. Fleas were grouped according to the breed, species and sex of the host. The effects of animal-level factors and geographical climate data on *R. felis* positivity in fleas were initially analyzed using univariate analysis using the *epistats* and *epiR* packages [17, 18].

Multivariable analyses were performed using the *glm* package [16], using factors with a *P*-value of less than or equal to 0.2 on univariate analysis and backwards elimination. Graphics were generated with *ggplot2* [19]. Map data were obtained from the GADM database.

## Results

Two hundred and twenty-five animals had valid, linkable location data available. In total, 488 fleas originating from 240 animals (cats and dogs) were identified and subjected to *R. felis* screening. All fleas were morphologically identified as *C. felis felis*.

**Fig. 1:**
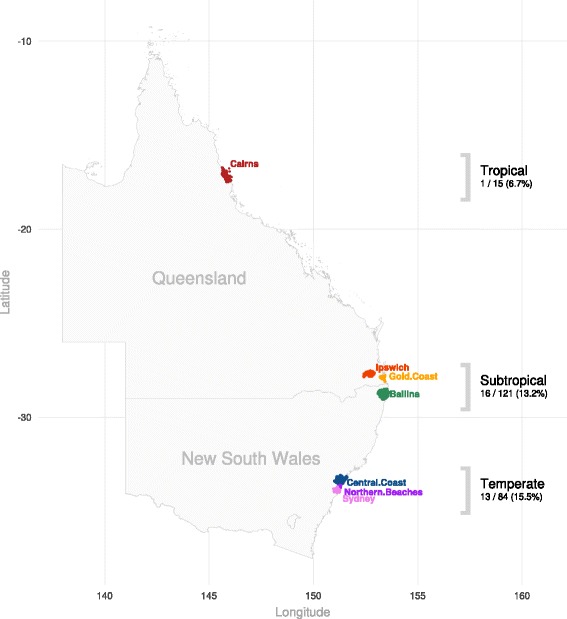
Collection regions and number of positive animals within each climatic zone

Rickettsial positivity within fleas sourced from the tropical, subtropical and temperate regions was noted in 6.7% (1/15), 13.2% (16/121) and 15.5% (13/84), respectively (Fig. 1). In total, fleas from 29 animals tested positive to *R. felis* by PCR. All isolates were identified as being 100% homologous to *R. felis* URRWXCal2 (GenBank: CP000053.1) by DNA sequencing at the *glt*A and *omp*B genes.

On univariate analysis, toy/small breed dogs were found to have a significantly reduced risk of harboring *R. felis-*positive fleas (*P* = 0.033) relative to large breed dogs. Pedigree breed and domestic medium hair (DMH) cats were also at a significantly reduced odds of exposure relative to domestic short hair cats (*P* = 0.0002 and *P* = 0.043, respectively) (Table 2). No other significant host or demographic factors were found to be related to *R. felis* positivity in fleas.

**Table 2:** Univariate analysis of animal factors on *R. felis* in fleas

	Population	Exposed	*P*	OR (95% CI)
Breed (cats)				
DSH	94	21	Ref.	1 (na–na)
DMH	24	1	0.043	0.151 (0.019–1.186)
DLH	3	0	1.000	0 (0–NaN)
Pedigree breed cat	46	0	< 0.0001	0 (0–NaN)
Breed (dogs)				
Large breed dog	65	14	Ref.	1 (na–na)
Toy/small breed dog	47	3	0.033	0.248 (0.067–0.921)
Species				
Cats	167	22	Ref.	1 (na–na)
Dogs	112	17	0.725	1.179 (0.595–2.337)
Sex				
Female	152	24	Ref.	1 (na–na)
Male	127	15	0.388	0.714 (0.357–1.429)
Region				
Ballina	87	19	Ref.	1 (na–na)
Central Coast	108	15	0.184	0.577 (0.274–1.217)
Cairns	12	1	0.450	0.325 (0.039–2.682)
Gold Coast	4	0	0.576	0 (0–NaN)
Ipswich	41	2	0.020	0.184 (0.041–0.83)
Northern Beaches	9	0	0.197	0 (0–NaN)
Climate				
Subtropical	132	21	Ref.	1 (na–na)
Temperate	117	15	0.589	0.777 (0.38–1.589)
Tropical	12	1	0.693	0.481 (0.059–3.922)
Temperature (daily minimum, °C)				
< 15	27	4	0.188	3.594 (0.747–17.303)
15–20	187	32	0.012	4.267 (1.26–14.445)
20–25	65	3	Ref.	1 (na–na)
Temperature (daily maximum, °C)				
< 27	118	12	Ref.	1 (na–na)
27–30	129	26	0.034	2.23 (1.068–4.654)
> 30	32	1	0.301	0.285 (0.036–2.278)

*Abbreviations*: na, not applicable, NaN, not a number, *P*, *P*-value, Ref., reference

**Fig. 2:**
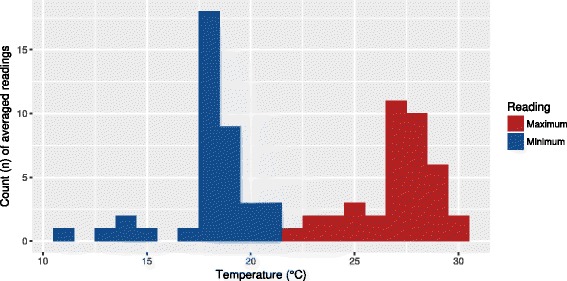
Distribution of minimum and maximum temperatures amongst positive fleas

Minimum average temperatures for the geographical regions *R. felis*-positive fleas were associated with (mean = 17.950 °C, SD = 2.089 °C) were normally distributed (Fig. 2) and significantly lower than the regions *R. felis*-negative fleas were associated with (mean = 18.795 °C, SD = 2.895 °C) on a Welch two sample t-test (*t*_(64.4)_ = -2.202, *df* = 64.425, *P* = 0.031). Maximum average temperatures of regions associated with positive fleas (mean = 27.036 °C, SD = 1.960 °C) were not significantly different from that of negative fleas (mean = 27.101 °C, SD = 2.840 °C).

**Fig. 3:**
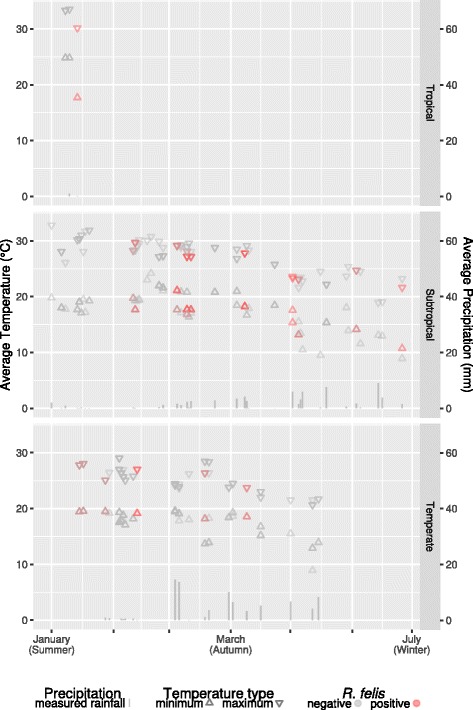
Average daily minimum and maximum environmental temperatures and precipitation for the 7-day period preceding collection of individual fleas

While no statistically significant geographical influence was noted in the univariate analysis, a dissimilar temporal distribution was seen in the 7-day temperature readings associated with positive fleas across subtropical and temperate regions (Fig. 3). In subtropical regions, there were relatively few fleas infected with *R. felis* during the warmer summer months. In comparison, infected fleas in temperate regions were noted throughout summer and autumn months, only dropping with the onset of colder winter temperatures.

This was further substantiated by multivariable regression modeling in which the odds of *R. felis* positivity in fleas was significantly more likely when minimum average environmental temperature fell within the 15–20 °C range (OR = 6.166, 95% CI = 2.012–26.910, *Z* = 2.840, *P* = 0.005) or below 15 °C (OR = 6.449, 95% CI = 1.223–37.716, *Z* = 2.201, *P* = 0.028) compared with a warmer minimum average temperature range of between 20–25 °C (Table 3). Concurrently, daily maximum temperatures between 27–30 °C correlated to higher odds of *R. felis* positivity in fleas (OR = 3.418, 95% CI = 1.1.603–7.649, *Z* = 3.106, *P* = 0.002) (Table 3).

**Table 3:** Multivariable regression modelling for environmental temperature on the prevalence of *R. felis* in fleas

	Population	Exposed	Coefficient (SE)	*Z*	*P*	OR (95% CI)
(Intercept)			-3.982 (0.709)	-5.621	0	0.019 (0.004–0.066)
Temperature (daily minimum, °C)						
< 15	27	4	1.864 (0.847)	2.201	0.028	6.449 (1.223–37.716)
15–20	187	32	1.819 (0.641)	2.84	0.005	6.166 (2.012–26.910)
20–25	65	3	Reference			
Temperature (daily maximum °C)						
< 27	118	12	Reference			
27–30	129	26	1.229 (0.396)	3.106	0.002	3.418 (1.603–7.649)
> 30	32	1	-0.757 (1.077)	-0.703	0.482	0.469 (0.025–2.653)

*Abbreviations*: *P*, *P*-value, *SE* standard error, *Z Z*-value

## Discussion

*Rickettsia felis* was found in fleas collected from cats and dogs across three different climatic regions of the eastern Australian coast, with the proportion of *R. felis*-positive flea-ridden animals reflecting previous studies [10].

All fleas were morphologically identified as *C. felis felis*, and all rickettsial DNA detected (*n* = 29) within these fleas was characterized as *R. felis* URRWXCal2. This study supports previous findings hypothesizing an association between *Rickettsia felis* URRWXCal2 and *C. felis felis*.

*Rickettsia felis* URRWXCal2 has been the primary subspecies documented to cause the clinical condition known as FBSF in humans [6]. As *C. felis felis* is the dominant flea in Australia, the potential public health threat presented by *R. felis* URRWXCal2 is of concern. Cases already attributable to FBSF have been noted in Australia [8, 20] as has evidence of prior exposure in asymptomatic persons knowingly or unknowingly in-contact with cat fleas [13].

Univariate analysis (Table 2) was suggestive that toy/small breed dogs had a lower odds of hosting *R. felis-*positive fleas relative to large breed dogs. Of the cats, DMH and pedigree breed cats had a lower odds compared to DSH cats. These animal-level factors are interesting findings that by themselves would be unlikely to drive changing presence of *R. felis* in hosted fleas. They may, however, be an indicator on potentially significant exposures that were not able to be quantified with this study: for example, the activity of the animal, living arrangements (indoor or outdoor), or time spent in environments where fleas are present. In isolation, there did not appear to be any statistically significant association of the climate category, species or sex of the animal on *R. felis* positivity in fleas.

Observing the distribution of local temperatures across the three climatic zones suggests there was a pattern to the occurrence of positive fleas - for warmer subtropical regions, the proportion of samplings for which an *R. felis*-positive flea was observed increased as temperatures trended downwards towards the winter months. Conversely, in cooler temperate regions, the proportion of *R. felis*-positive fleas increased towards the warmer summer months.

A significant difference in minimum average temperature for the week preceding sampling of positive fleas (mean = 17.951 °C, SD = 2.089 °C) was noted compared to the minimum average temperature over the week preceding sampling of negative fleas (mean = 18.795 °C, SD = 2.895 °C). Multivariable modeling was suggestive that minimum and maximum environmental temperature ranges were significant predictors (Table 3). Relatively low average minimum daily temperature ranges of 15–20 °C (OR = 6.166, 95% CI = 2.012–26.910, *Z* = 2.840, *P* = 0.005) and below 15 °C (OR = 6.449, 95% CI = 1.223–37.716, *Z* = 2.201, *P* = 0.028), had an increased odds of *R. felis* positivity in fleas compared with the 20–25 °C range. Average maximum daily temperature showed an effect where a constrained interval of 27–30 °C was associated with an increased odds of *R. felis* infection within fleas (OR = 3.418, 95% CI = 1.603–7.649, *Z* = 3.016, *P* = 0.002).

*Rickettsia felis* is known to be preferentially cultured at 28 °C rather than 34 °C typical of other rickettsiae [21], making these findings consistent with its theoretical ability to survive and thrive within these fleas. Its persistence at cooler minimum environmental temperatures within the vector host suggests that this bacteria is tolerant of cold temperature periods; conversely warmer temperatures lead to less prevalence. Cat fleas can spend substantial periods of their life-cycle in the environment or prolonged periods as a permanent ectoparasite (in excess of 113 days) on the animal [22], where local environmental temperatures may suit *R. felis* growth and maintenance within the flea.

These results support our previous findings, where exposure of Australian veterinarians was found to be most common in the cooler temperate states of Victoria and Tasmania, and demonstrates that in Australia *R. felis* positivity within *C. felis felis* appears to be environmentally dependent [13].

More studies in other countries are needed to determine if these findings are applicable to the life-cycle of *R. felis* URRWXCal2 globally. Evidence of the organism or exposure to the organism has been widely reported, including within temperate parts of the world [23]. Its presence in cooler regions in Australia is complementary to previous findings of closely related rickettsial species such as *R.* RF2125 in tropical-subtropical climates and different vectors [2, 3]. Nevertheless, tolerance to a wide spectrum of environmental conditions is likely to play a beneficial role in allowing *R. felis* URRWXCal2 to infect fleas across regions and continents and throughout seasonal temperature variation.

The findings of this study suggest that environmental factors can potentially act as predictors for zoonotic vector-borne disease risk, particularly for those transmitted by arthropods with off-host portions of their life-cycle. Awareness of flea-borne diseases is inconsistent, even in veterinary workers [13]. Given the propensity for *R. felis* URRWXCal2 to persist in fleas during cooler environmental conditions, flea prophylaxis coverage should be consistently maintained even across winter periods especially in subtropical climates.

## Conclusions

Environmental temperature appears to influence the prevalence of *R. felis* in its flea vector host. The relationship of *R. felis* in the cat flea at cooler temperatures suggests that maintaining flea control during winter months should be a priority for cats and dogs to reduce their exposure to infected fleas, thus limiting potential human exposure.

## Abbreviations

ARRL: Australian Rickettsial Reference Laboratory
CFT: Cat flea typhus
DLH: Domestic long hair
DMH: Domestic medium hair
DNA: Deoxyribonucleic acid
DSH: Domestic short hair
FBSF: Flea-borne spotted fever
GADM: Global administrative areas
*glt*A: Citrate synthase gene
*omp*B: Outer membrane protein B
PBS: Phosphate buffered saline
PCR: Polymerase chain reaction
qPCR: Real-time PCR
SD: Standard deviation
SE: Standard error
